# His-Bundle Pacing in a Patient with Transcatheter Aortic Valve Implantation-Induced Left Bundle Branch Block

**DOI:** 10.1155/2018/4606271

**Published:** 2018-08-23

**Authors:** Jonathan Sen, Michael Mok, Mark Perrin

**Affiliations:** University Hospital Geelong, Barwon Health, PO Box 281, Geelong, VIC 3220, Australia

## Abstract

Transcatheter aortic valve implantation (TAVI) is an effective intervention for severe aortic stenosis in patients at intermediate or high surgical risk, but damage to the native conduction system such as left bundle branch block (LBBB) may offset its benefits. New onset LBBB is associated with a higher risk of cardiovascular morbidity and mortality. His-bundle pacing (HBP) may be useful to treat TAVI-induced LBBB but has yet to be reported. We present the case of a 76-year-old man with severe symptomatic aortic stenosis treated with TAVI. His preoperative electrocardiogram showed sinus rhythm with a narrow QRS complex. Insertion of a CoreValve Evolut R transcatheter aortic valve was uneventful apart from the development of LBBB with a long PR interval. A dual-lead DDD pacemaker was implanted via the left cephalic vein on the following day. HV was mildly prolonged at 60 ms. Capture of the proximal His restored AV synchrony without correction of LBBB. Repositioning of the lead with capture of the left bundle branch enabled complete ventricular resynchronisation with a single lead. Our case demonstrates that LBBB in the setting of TAVI may be corrected by HBP.

## 1. Introduction

Transcatheter aortic valve implantation (TAVI) is an effective treatment for severe aortic stenosis in patients at intermediate or high surgical risk [1, 2]. However, damage to the native conduction system including complete atrioventricular (AV) block (6–25%) and new left bundle branch block (LBBB) (4–65%) may occur and offset the benefit of the intervention [3–5]. New-onset LBBB causes a delay in depolarisation of the left ventricle and predicts higher cardiovascular morbidity and mortality and high rates of pacemaker implantation [2, 6].

Pacing at the distal His bundle may correct LBBB. Case reports support the concept of longitudinal dissociation in the His bundle [7], that is, fibres within the His bundle are predestined for the left or right bundle branch. Pacing at the His bundle may capture fibres of a bundle branch distal to the site of intra-Hisian block [6]. However, to our knowledge, the correction of TAVI-induced LBBB via HBP has not been reported [7, 8]. This is likely due to more distal block and the relative inaccessibility of the left bundle branch conduction fibres to the pacing lead helix. Here, we describe HBP as a therapeutic intervention for patients with TAVI-induced LBBB.

## 2. Case Presentation

A 76-year-old man was electively admitted for intervention and management of severe symptomatic aortic stenosis resulting in worsening New York Heart Association Class III cardiac failure. His medical history included stage III chronic kidney disease, type 2 diabetes mellitus, hypertension, and prior coronary artery bypass grafting. Coronary angiography demonstrated a patent left internal mammary artery graft to the left anterior descending coronary artery and a saphenous vein graft to the dominant distal left circumflex artery with a severe stenosis distal to the surgical anastomosis.

Transthoracic echocardiography was performed and showed a thickened and calcified aortic valve with reduced cusp excursion, mild concentric left ventricular hypertrophy with normal left ventricular cavity size, and systolic function. The left atrium was severely dilated. Left ventricular ejection fraction was above 55%. Valve area was estimated at 0.8 cm^2^, with a measured mean gradient of 44 mmHg.

A cardiac conference was held to discuss intervention for his severe aortic stenosis. TAVI was chosen in preference to a redo sternotomy in the setting of the Society of Thoracic Surgeons score of 5.8% (intermediate risk cardiac surgery), stable coronary artery disease, and in accordance with the patient's preference.

**Figure 1:**
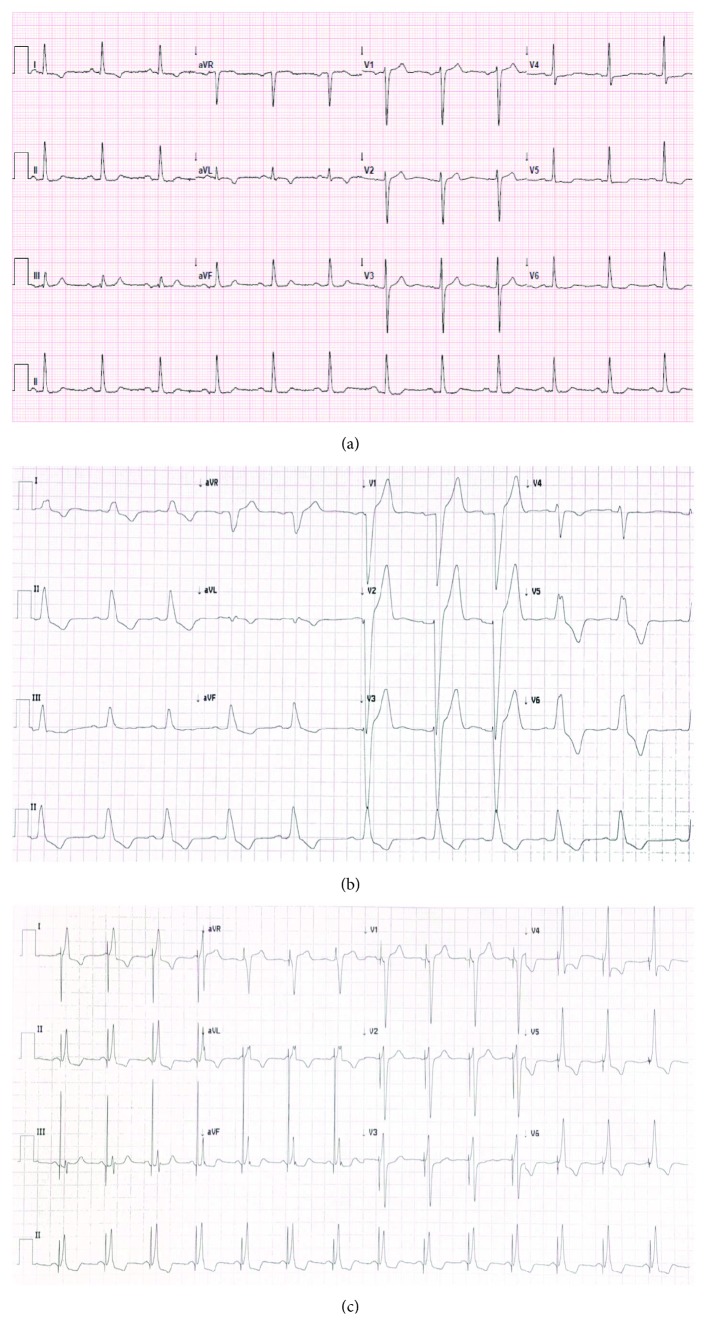
Electrocardiogram tracings at different time points. (a) Pre-transcatheter aortic valve implantation; (b) prolonged PR interval with left bundle branch block post-transcatheter aortic valve implantation; (c) post-His-bundle pacing with correction of the left bundle branch block.

The preoperative electrocardiogram (ECG) showed sinus rhythm with a narrow QRS complex (Figure 1(a)). Using a right femoral approach, a CoreValve Evolut R 29 mm (Medtronic, Minneapolis, Minnesota) transcatheter aortic valve was deployed after balloon aortic valvuloplasty with an 18 mm Cristal balloon. TAVI was uneventful. Postdilatation was performed using a 23 mm Cristal balloon due to moderate paravalvular aortic regurgitation around the left coronary cusp seen on a postprocedure transoesophageal echocardiogram. At the time of TAVI, the patient developed LBBB (average QRS duration of 180 ms) with a prolonged PR interval of 240 ms (Figure 1(b)). Within the first 24 hours post-TAVI, he also had episodes of high-grade AV block.

**Figure 2:**
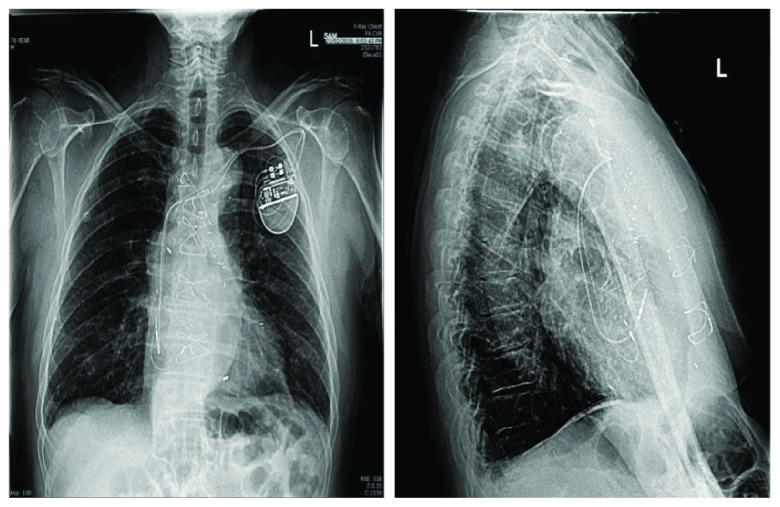
Chest X-rays (posterior-anterior and lateral views) after transcatheter aortic valve implantation and His-bundle pacemaker insertion.

A dual-lead Boston Scientific Accolade™ Extended Life DR Pacemaker was implanted via the left cephalic vein (Figure 2). The HV interval was mildly prolonged at 60 ms. Proximal His capture (selective) threshold was less than 1 V without recruitment of the left bundle branch; RV myocardial capture threshold was 1.5 V at 1 ms; correction of LBBB—due to the presumed capture of distal His bundle and recruitment of the left bundle branch—occurred at 4.5-5 V at 1 ms. A Medtronic 5076 lead was then placed in the right atrial appendage with a threshold less than 1 V. The device was programmed DDD 50. The paced QRS duration on 12 lead ECG was 125 ms consistent with nonselective HBP (para-Hisian morphology) (Figure 1(c)). At 1 month, 12 lead electrocardiogram tracing showed continued correction of LBBB at 5 V @ 1 ms.

## 3. Discussion

TAVI is an increasingly common intervention for patients with severe symptomatic aortic stenosis at intermediate or high risk with standard surgery. The multinational randomized Surgical Replacement and Transcatheter Aortic Valve Implantation trial (SURTAVI) demonstrated that TAVI was noninferior to surgical replacement based on a composite of all-cause mortality or disabling stroke at 2 years; however, TAVI had markedly higher rates of pacemaker implantation [9].

The risk of heart block and the need for permanent pacing is higher with CoreValve (25%) than other TAVI models [10, 11]. The CoreValve's self-expanding frame and deeper implantation into the left ventricular outflow tract may result in mechanical injury of the His bundle and/or left bundle branch accounting for this higher risk [11].

LBBB is the most common TAVI-induced conduction abnormality reported in 29–65% of patients implanted with CoreValve [11, 12]. LBBB causes rhythmic and haemodynamic complications, is associated with worse outcomes, and can lead to heart failure [13]. The effect of new-onset LBBB on mortality is uncertain, but some studies found a significantly higher mortality rate in patients who develop LBBB post-TAVI [11, 14]. Even though the incidence of new-onset LBBB is higher in patients implanted with CoreValve compared to Edwards SAPIEN valve, no difference in mortality was observed [14].

There is no published guideline on the management of LBBB post-TAVI. In our case, we elected to place a pacemaker due to the presence of a long PR interval, new-onset LBBB, and an expanding valve. The His bundle contains fibres predestined to form the right and left bundle branches, a theory confirmed by correction of bundle branch block by pacing at the His bundle presumably due to the recruitment of fibres distal to the site of block [15]. In our case, these fibres were found by mapping for the His bundle potential, with small movements of the lead tip until a suitable pacing site where correction of the underlying LBBB was achieved, albeit at high pacing output.

A standard dual-chamber device would resynchronise the atrial and ventricular chambers, but leave the ventricular chambers dyssynchronous. We placed a dual-chamber His bundle device to allow resynchronisation of all chambers. In the largest series to date of HBP in patients with heart block postprosthetic valve replacement, Sharma and colleagues included four patients post-TAVI—all Edward SAPIEN valves: in two cases, HBP was unsuccessful and a standard right ventricular lead was placed—in the remaining two patients, HBP was employed successfully to correct RBBB in one case and to retain a narrow QRS complex in the other [8]. In our case, we were able to obtain selective HBP at a low threshold, but true correction of LBBB—presumably due to more distal disease—was only possible at higher output.

The tools for HBP are rudimentary. The Medtronic SelectSecure lead has an exposed helix screw with no inner lumen and must be delivered through a fixed sheath (Medtronic 315His) delivered through a 7 French introducer sheath, or a deflectable sheath (Medtronic SelectSite C304) delivered through a 9Fr introducer sheath. Implantation is therefore limited by anatomy. It may be that improved delivery tools allow more precise placement of the lead with lower subsequent thresholds. To some extent, the higher output required in our case may be mitigated through device programming by increased pulse width, pacing with output marginally (0.5-1 V) above the left bundle branch threshold, and the use of a pacemaker with an extended-life battery.

## 4. Conclusion

Our case demonstrates that LBBB in the setting of TAVI may be corrected by pacing at the His bundle. To our knowledge, this is the first report of HBP with a self-expanding valve and the first report for left bundle branch recruitment in this setting. HBP allows complete AV and ventricular resynchronisation with two leads. Longer-term follow-up will be needed to determine the chronic threshold of LBBB correction.

## References

[1] Pighi M., Serdoz R., Kilic I. D., Sherif S. A., Lindsay A., di Mario C. (2014). TAVI: new trials and registries offer further welcome evidence - U.S. CoreValve, CHOICE, and GARY. *Global Cardiology Science and Practice*.

[2] Calvi V., Pruiti G. P. (2014). Pacemaker implantation and need for ventricular pacing during follow-up after transcatheter aortic valve implantation. *Pacing and Clinical Electrophysiology*.

[3] Kostopoulou A., Karyofillis P., Livanis E. (2016). Permanent pacing after transcatheter aortic valve implantation of a CoreValve prosthesis as determined by electrocardiographic and electrophysiological predictors: a single-centre experience. *Europace*.

[4] Engels E. B., Poels T. T., Houthuizen P. (2016). Electrical remodelling in patients with iatrogenic left bundle branch block. *Europace*.

[5] Sundh F., Ugander M. (2014). Impact of left bundle branch block after transcatheter aortic valve replacement. *Journal of Electrocardiology*.

[6] Teng A. E., Lustgarten D. L., Vijayaraman P. (2016). Usefulness of His bundle pacing to achieve electrical resynchronization in patients with complete left bundle branch block and the relation between native QRS Axis, duration, and normalization. *The American Journal of Cardiology*.

[7] Sharma P. S., Ellison K., Patel H. N., Trohman R. G. (2017). Overcoming left bundle branch block by permanent His bundle pacing: evidence of longitudinal dissociation in the His via recordings from a permanent pacing lead. *HeartRhythm Case Reports*.

[8] Sharma P. S., Subzposh F. A., Ellenbogen K. A., Vijayaraman P. (2017). Permanent His-bundle pacing in patients with prosthetic cardiac valves. *Heart Rhythm*.

[9] Reardon M. J., van Mieghem N., Popma J. J. (2017). Surgical or Transcatheter aortic-valve replacement in intermediate-risk patients. *New England Journal of Medicine*.

[10] Nazif T. M., Dizon J. M., Hahn R. T. (2015). Predictors and clinical outcomes of permanent pacemaker implantation after transcatheter aortic valve replacement: the PARTNER (Placement of AoRtic TraNscathetER Valves) trial and registry. *JACC: Cardiovascular Interventions*.

[11] Young Lee M., Chilakamarri Yeshwant S., Chava S., Lawrence Lustgarten D. (2015). Mechanisms of heart block after Transcatheter aortic valve replacement – cardiac anatomy, clinical predictors and mechanical factors that contribute to permanent pacemaker implantation. *Arrhythmia & Electrophysiology Review*.

[12] van der Boon R. M., Nuis R. J., van Mieghem N. M. (2012). New conduction abnormalities after TAVI--frequency and causes. *Nature Reviews Cardiology*.

[13] Massoullié G., Bordachar P., Irles D. (2016). Prognosis assessment of persistent left bundle branch block after TAVI by an electrophysiological and remote monitoring risk-adapted algorithm: rationale and design of the multicentre LBBB–TAVI study. *BMJ Open*.

[14] Schymik G., Tzamalis P., Bramlage P. (2015). Clinical impact of a new left bundle branch block following TAVI implantation: 1-year results of the TAVIK cohort. *Clinical Research in Cardiology*.

[15] Narula O. S. (1977). Longitudinal dissociation in the His bundle. Bundle branch block due to asynchronous conduction within the His bundle in man. *Circulation*.

